# *Caenorhabditis elegans* Extracts Stimulate IAA Biosynthesis in *Arthrobacter pascens* ZZ21 via the Indole-3-pyruvic Acid Pathway

**DOI:** 10.3390/microorganisms9050970

**Published:** 2021-04-30

**Authors:** Mengsha Li, Teng Li, Ming Zhou, Mengdi Li, Yexin Zhao, Jingjing Xu, Feng Hu, Huixin Li

**Affiliations:** 1 College of Resources and Environmental Sciences, Nanjing Agricultural University, Nanjing 210095, China; limonms@163.com (M.L.); liteng035@njau.edu.cn (T.L.); 2018103074@njau.edu.cn (M.Z.); 2018103081@njau.edu.cn (M.L.); zhaoyexin@njau.edu.cn (Y.Z.); xujingjing@njau.edu.cn (J.X.); fenghu@njau.edu.cn (F.H.); 2 College of Science & Technology, Ningbo University, Cixi 315300, China; 3 Jiangsu Collaborative Innovation Center for Solid Organic Waste Resource Utilization, Nanjing 210014, China

**Keywords:** *C. elegans* extracts, IAA-producing bacteria, IAA, indole-3-pyruvic acid pathway, pyruvate, NADH

## Abstract

Inter-organismal metabolites play important roles in regulating organism behavior and the communication between organisms. Nematodes, the most abundant animals on earth, are crucial participants in soil ecosystems through their interactions with microbes. For example, bacterial-feeding nematodes increase the activity of indole-3-acetic acid (IAA)-producing bacteria and the IAA content in soil. However, the way in which these nematodes interact with bacteria and affect IAA biosynthesis is not well understood. Here, using the model nematode *Caenorhabditis elegans* and the plant-beneficial bacterium *Arthrobacter pascens* ZZ21, we examined the effects of nematode excretions or extracts on bacterial IAA biosynthesis. To explore the underlying regulatory mechanism in more detail, we performed transcriptome sequencing and metabolomic analysis. Our findings suggest that *C. elegans* extracts promote IAA biosynthesis in *A. pascens* ZZ21 by increasing the expression of genes and the abundance of intermediates involved in the indole-3-pyruvic acid (IPyA) pathway. *C. elegans* extracts also significantly influenced biosynthetic and metabolic activity in *A. pascens* ZZ21. Treatment with *C. elegans* extracts promoted pyruvate metabolism, the citrate cycle (TCA) cycle and the production of some TCA-cycle-related amino acids and inhibited oxidative phosphorylation, which induced the accumulation of reduced nicotinamide adenine dinucleotide (NADH). We propose that the extracts altered the metabolism of *A. pascens* ZZ21 to help the bacteria resist stress caused by their predator. Our findings indicate that bacterial-feeding nematodes mediate the interaction between nematodes and bacteria via their extracts, providing insights into the ecological function of *C. elegans* in soil.

## 1. Introduction

Inter-organismal metabolites function as messengers that help regulate the behaviors and physiological processes of many organisms, especially in soil ecosystems, which harbor diverse populations of bacteria, fungi, nematodes, protists and plants [1,2,3,4]. Metabolites released by soil organisms play important roles in maintaining proper soil conditions and other physical parameters to aid these organisms in the face of environmental stress. For example, plants produce specialized metabolites that modulate the root microbiota by attracting, inhibiting or even killing soil microorganisms, thereby promoting plant growth and defense against pathogens [5,6,7,8,9]. In turn, bacterial exudates can either promote plant infection by pathogens [10] or act as antibiotics that are antagonistic to pathogens [11,12]. Hence, understanding the interactions between soil organisms will have considerable utility for maintaining a stable soil system and further increasing crop yields.

Nematodes, one of the major functional groups in soil, play a variety of roles in the ecosystem due to their diverse feeding habits [13,14]. Bacterial-feeding nematodes are the most abundant type of nematodes in soil. These nematodes influence the quantity, activities and communities of soil microbes via their metabolic activities and feeding behaviors, thereby affecting nutrient transformation and circulation and regulating plant growth and development [15,16,17,18,19]. We previously demonstrated that bacterial-feeding nematodes increased the activities of bacteria and the levels of indole-3-acetic acid (IAA) in the soil [20,21,22,23]. To our knowledge, bacterial-feeding nematodes do not have the ability to produce IAA, so it is reasonable to attribute the increase in IAA levels in soil to interactions between nematodes and IAA-producing bacteria. However, the way in which bacterial-feeding nematodes interact with IAA-producing bacteria and the way in which nematodes modulate bacterial IAA biosynthesis are not fully understood. Several studies have demonstrated that various plant metabolites promote IAA production in bacteria. For example, Prinsen et al. [24] and Theunis et al. [25] demonstrated that the flavonoids produced by host plants that accumulate in the rhizosphere caused the root-symbiotic bacterium *Rhizobium* spp. to synthesize IAA. Jasim et al. [26] reported that treatment with metabolites (piperine and other compounds) extracted from *Piper nigrum* stimulated endophytic bacteria to produce IAA. Similarly, nematodes secrete many metabolites, including amino acids, sugars and organic acids, which play important ecological roles in the soil system [27,28,29]. Some metabolites are essential in maintaining the balance of the soil ecosystem, for example, by regulating the behavior of nematodes, eliciting plant defense responses and functioning in pathogen resistance [7,30,31,32]. Therefore, we hypothesized that bacterial-feeding nematodes regulate IAA biosynthesis in bacteria via their secretory metabolites.

**Figure 1:**
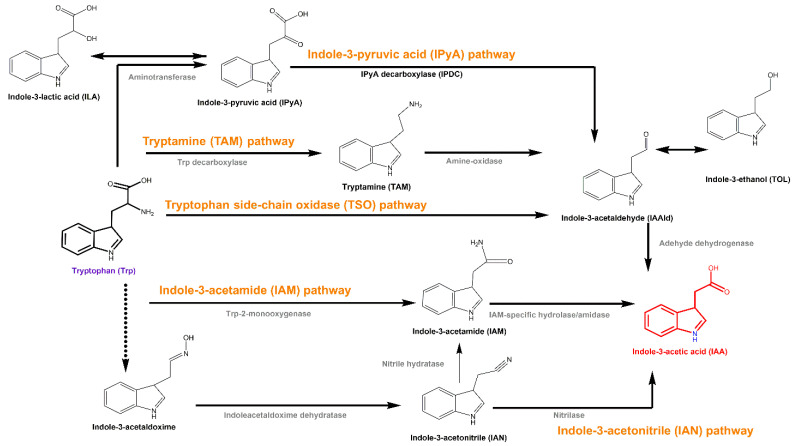
Tryptophan-dependent biosynthesis pathways. Trp: tryptophan; IAA: indole-3-acetic acid; IAM: indole-3-acetamide; IAN: indole-3-acetonitrile; TAM: tryptamine; IPyA: indole-3-pyruvic acid; ILA: indole-3-lactic acid; IAAld: indole-3-acetaldehyde; TOL: indole-3-ethanol.

IAA is the most common naturally occurring phytohormone that regulates plant growth and development [33,34,35]. It also serves as a signal in communication and interaction among soil organisms [36,37,38]. Most plant-beneficial bacteria can produce IAA. Tryptophan is a major precursor for bacterial IAA biosynthesis [39], and five tryptophan-dependent biosynthesis pathways have been described in detail—the indole-3-acetamide (IAM), indole-3-pyruvic acid (IPyA), indole-3-acetonitrile, tryptamine and tryptophan side-chain oxidase pathways (Figure 1), which are named based on their distinctive intermediates [40]. The plant-beneficial bacterium *Arthrobacter pascens* strain ZZ21 synthesizes IAA via the IAM and IPyA pathways [41]. Six genes related to IAA biosynthesis are present in *A. pascens* ZZ21—*iaaM* (predicted to encode tryptophan 2-monoxygenase), *aam* and *gatA* (both predicted to encode amidases) in the IAM pathway; and *prr*, *puuC* and *aldH* (predicted to encode aldehyde dehydrogenase) in the IPyA pathway [41]. In addition, three intermediates (IAM, IPyA and its enzymatic reduction product indole-3-lactic acid) are involved in the IAA biosynthesis pathways.

**Figure 2:**
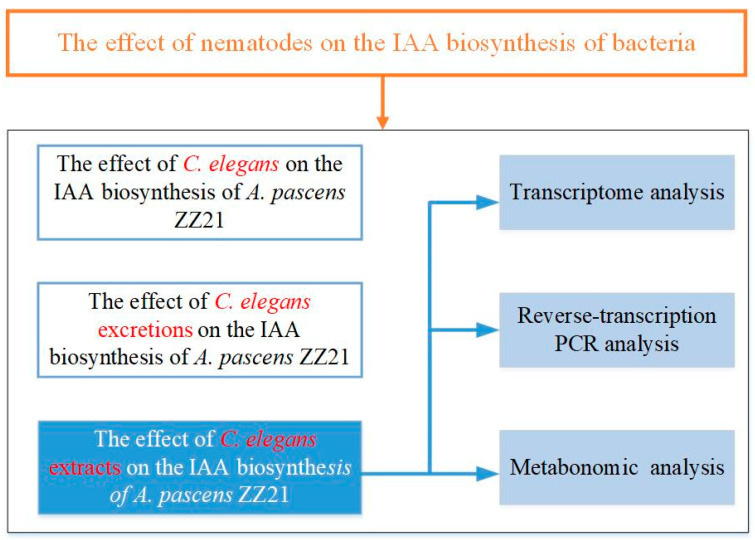
Flowchart of the experimental design.

The objective of this study was to determine whether metabolites produced by bacterial-feeding nematodes influence IAA biosynthesis in bacteria and to explore the underlying regulatory mechanism (the experimental design is shown in Figure 2). We tested the effects of the model nematode *Caenorhabditis elegans* on IAA biosynthesis in the plant-beneficial bacterium *A. pascens* ZZ21 grown in liquid mineral medium, including the effects of its secreted excretions or extracts. We then performed transcriptome and metabolome analyses to investigate how *C. elegans* extracts promote IAA biosynthesis in bacteria. We also explored the effects of *C. elegans* extracts on other metabolic processes in the bacterium via transcriptome and metabolome analysis.

## 2. Materials and Methods

### 2.1. Preparation of Nematodes, Secreted Excretions and Extracts

*C. elegans* N2 was obtained from the Caenorhabditis Genetics Center. The nematodes were cultivated at 20 °C on Petri dishes containing freshly prepared nematode growth medium (3 g NaCl, 2.5 g peptone, 17 g agar and 975 mL H_2_O; the medium was autoclaved, cooled to 55 °C, and supplied with 25 mL 1 M KPO_4_ buffer (pH 6.0), 1 mL 1 M CaCl_2_, 1 mL 1 M MgSO_4_ and 1 mL 5 mg/mL cholesterol in ethanol that had been filtered through a 0.22-μm filter). After reaching the desired stage, the worms were harvested from the Petri plates in M9 buffer (5 g NaCl, 3 g KH_2_PO_4_, 6 g Na_2_HPO_4_, 1 mL 1 M MgSO_4_, H_2_O to 1 L, autoclaved) and collected using the modified Baermann funnel method [42]. To remove the medium and bacteria from the nematode cuticles, the worms were washed with M9 buffer and centrifuged at 22 °C at 1500 g for 3 min; this process was repeated three times. The nematodes were incubated in M9 buffer for 30 min at 22 °C with shaking at 250 rpm to digest the bacteria in their guts. After being washed three times with sterile water, the worms were collected for further experiments [29]. All operations were performed under aseptic conditions.

To collect the excretions secreted by the nematodes, the worms were incubated in sterile water for 1 h at 22 °C with shaking at 250 rpm at a density of ~15,000 worms/mL. Excretions were collected from the worms by means of gentle centrifugation at 1500 g, at 22 °C, for 5 min, filtered through a 0.22-μm filter, lyophilized and stored at −80 °C. The excretions secreted by one worm in 1 h are defined as 1 worm equivalent (WE) [29]. To collect nematode extracts, the worms were incubated in sterile water for 1 h at 22 °C with shaking at 250 rpm and broken up with an Ultrasonic Cell Crusher (XO-1000D) with a 60% duty ratio (working for 3 s and resting for 3 s) for 45 min. The extracts were collected by means of centrifugation at 1500 g, at 22 °C, for 5 min, filtered through a 0.22-μm filter, lyophilized and stored at −80 °C.

### 2.2. Isolation of Bacteria and Culture Conditions

The IAA-producing bacterium *A. pascens* ZZ21 (China General Microbiology Culture Collection Center, CGMCC accession no. 7325) was isolated from forest soil from Zijin Mountain in Nanjing City, Jiangsu Province, China. The bacteria were cultured in liquid mineral medium (5 g glucose, 2 g (NH_4_)_2_SO_4_, 0.5 g NaH_2_PO_4_, 0.5 g K_2_HPO_4_, 0.2 g MgSO_4_·7H_2_O and 0.1 g CaCl_2_·2H_2_O with 1 L H_2_O at pH 7.0) at 30 °C with shaking at 250 rpm. To stimulate bacterial IAA biosynthesis, 200 mg/L tryptophan was added to the culture medium.

### 2.3. Analysis of the Effects of C. elegans and Its Excretions or Extracts on Bacterial IAA Biosynthesis

Experiments were performed to evaluate IAA production by *A. pascens* ZZ21 under various conditions. The bacteria were cultured in 30 mL liquid mineral medium. To investigate the effects of nematodes on bacterial IAA biosynthesis, *C. elegans* was added to the medium at 80 adult worms/mL (based on the results of Jiang et al. [43]). The samples were cultured in an incubator set at 22 °C for 7 days (the optimal incubation temperature and time for nematode growth). The IAA concentration in the medium was measured on day 1, 2, 3, 5 and 7 of incubation. To test the effects of nematode-secreted excretions on bacterial IAA biosynthesis, *C. elegans*-secreted excretions were added to the medium at 2000 WE/mL (equivalent to the levels of excretions secreted by 80 worms for 1 day). The samples were transferred to an incubator set at 30 °C and cultured for 3 days, and IAA concentration in the medium was measured daily. To test the effects of nematode extracts on bacterial IAA biosynthesis, *C. elegans* extracts were added to the medium at 2000 WE/mL. The samples were cultured in an incubator set at 30 °C for 3 days and the concentration of IAA in the medium was measured daily. Samples without *C. elegans*, their excretions or extracts were used as controls.

### 2.4. Quantification of IAA Levels

IAA levels in the supernatants of samples were measured spectrophotometrically [44]. The supernatants were mixed at equal ratios with Salkowski reagent (50 mL 35% HClO_4_ combined with 1 mL 0.5 M FeCl_3_). After the samples were incubated in the dark for 30 min at room temperature for color development, the absorbance was measured at 530 nm with a spectrophotometer. The IAA concentration was calculated by comparing the absorbance with a standard curve constructed using known concentrations of IAA. The OD_600_ was determined at each time point.

### 2.5. Effects of C. elegans Extracts on Gene Expression in A. pascens ZZ21

#### 2.5.1. RNA Sequencing and Analysis

Total RNA was extracted from samples from each group (with three biological replications). RNA quality was assessed using an Agilent 2100 Bioanalyzer and NanoDrop. cDNA libraries were constructed using a SMART cDNA Library Construction Kit (TaKaRa, Dalian, China) according to the manufacturer’s instructions. The libraries were sequenced on an Illumina HiSeq 4000 platform, and 150 base pair (bp) paired-end reads were generated. Raw reads obtained by sequencing were cleaned by filtering with Trimmomatic software. After quality control with NGS QC Toolkit, the clean reads were mapped to the reference genome (the whole genome of *A. pascens* ZZ21) using Bowtie 2. The maximum number of allowed mismatched bases per read was set at 0; the other parameters were set as the software defaults. The expression levels of genes were measured by fragments per kilobase of the exon model per million mapped reads (FPKM). HTSeq software was used to analyze the gene expression levels of different samples. Differentially expressed genes of *A. pascens* ZZ21 under different treatments were identified (*p* < 0.05 and |log2(fold change)| > 1.5) and subjected to Kyoto Encyclopedia of Genes and Genomes (KEGG) enrichment analysis. KEGG pathways with *p* < 0.05 and containing more than five genes were defined as significantly enriched KEGG pathways.

#### 2.5.2. Reverse-Transcription PCR Analysis of Genes Involved in IAA Biosynthesis

To verify the effects of *C. elegans* extracts on the expression of genes involved in IAA biosynthesis, total RNA was extracted from *A. pascens* ZZ21 cultured in minimal liquid medium with or without *C. elegans* extracts. The RNA was reverse-transcribed into cDNA using a Reverse-transcription Reagent Kit (TaKaRa, Dalian, China) according to the manufacturer’s instructions. The resulting cDNA was diluted 1:100 in RNase-free (ribonuclease-free) water and used as template DNA for quantitative reverse-transcription PCR (qRT-PCR) with a SYBR Premix Ex Taq Kit (TaKaRa, Dalian, China). The reactions were carried out on an ABI Step One Plus Real-Time PCR system under the following conditions—3 min at 95 °C for denaturation, followed by 40 cycles of 10 s at 95 °C, 30 s at 55 °C and 20 s at 72 °C. The primers used for qRT-PCR are listed in Table S1. The transcript levels of genes involved in IAA biosynthesis were normalized using the copy number of the 16 s rDNA (ribosomal DNA) gene from *A. pascens* ZZ21. Gene expression levels were analyzed using the −∆∆Ct method.

### 2.6. Metabolite Analysis

Metabolites were collected from samples (with five biological replications per group) by gravity, filtered through a 0.22-µm filter, lyophilized and stored at −80 °C. The lyophilized samples were re-dissolved in 10 mL methanol-water (4:1, *v*/*v*), and then 20 μL of internal standard (l-2-chloro-phenylalanine, 0.3 mg/mL in methanol) was added to 1 mL of sample solution. After centrifugation for 10 min (12,000 rpm, 4 °C), 200 μL of supernatant was put into a glass vial and then evaporated with a freeze concentration centrifugal dryer. Eighty microliters of 15 mg/L methoxyamine hydrochloride pyridine solution was added to the vial. After vortexing for 2 min, the samples were loaded to a shaking incubator at 37 °C for 90 min for the oximation reaction. Then, 80 μL of bis(trimethylsilyl)trifluoroacetamide (1% chlorotrimethylsilane) derivatization reagent and 20 μL of n-hexane were added to the vial. After vortexing for 2 min, the samples were allowed to react at 70 °C for 60 min. Finally, the samples were left to stand for 30 min at room temperature prior to gas chromatography-mass spectrometry (GC-MS) metabolomics analysis. Quality control (QC) samples were prepared by mixing equal volumes of extracts from all samples, each at the same volume as the test sample.

An Agilent 7890B gas chromatography system, coupled to an Agilent 5977A MSD system (Agilent Technologies Inc., Santa Clara, CA, USA), was used to analyze the samples. An Agilent DB-5MS fused-silica capillary column (30 m × 0.25 mm × 0.25 μm, Agilent J&W Scientific, Folsom, CA, USA) was used for analysis. Helium (>99.999%) was used as the carrier gas, with an injection temperature of 260 °C and a flow rate of 1 mL/min. The injection volume was 1 μL with a split ratio of 10:1, and the solvent delay time was set to 5 min. The initial temperature of the column oven was 60 °C, which was increased to 125 °C at a rate of 8 °C/min, and then to 210 °C at 5 °C/min, to 270 °C at 10 °C/min, and to 305 °C at 20 °C/min, and finally held for 5 min. The temperatures of the ion source (electron impact) and MS quadrupole were 150 °C and 230 °C, respectively. The collision energy was set to 70 eV. Mass spectrometric data were collected in full-scan mode (*m/z* 50–450). To assess the repeatability of the entire analysis, a QC sample was inserted into 1 in every 10 analytical samples.

ChemStation (version E.02.02.1431, Agilent, Santa Clara, CA, USA) was used to convert the file format of the raw data from the apparatus to the common data format (CDF). ChromaTOF (version 4.34, LECO, St. Joseph, MI, USA) was used to analyze the data; we used *A. pascens* ZZ21 treated with *C. elegans* extracts as the experimental group, and compared them with the bacteria without the treatment with *C. elegans* extracts (*p* < 0.05, |log2(fold change)| > 0.6). Metabolites were subjected to qualitative analysis using the National Institute of Standards and Technology (NIST) and Fiehn databases.

### 2.7. Statistical Analysis

All experimental data were analyzed with the SPSS 22.0 statistics package (IBM). One-way ANOVA was performed to examine the effects of nematodes, their excretions or extracts on bacterial IAA biosynthesis and the expression of genes related to IAA biosynthesis (qRT-PCR), followed by the Duncan test (*p* < 0.05). Transcriptomic and metabolomic data were analyzed with R software. DESeq was used to analyze the differentially expressed genes, and partial least-squares discrimination analysis was used to analyze the differential metabolites of *A. pascens* ZZ21 treated with *C. elegans* extracts.

## 3. Results

### 3.1. A. pascens ZZ21 Shows an Increased Ability to Synthesize IAA in the Presence of C. elegans or Its Excretions or Extracts

**Figure 3:**
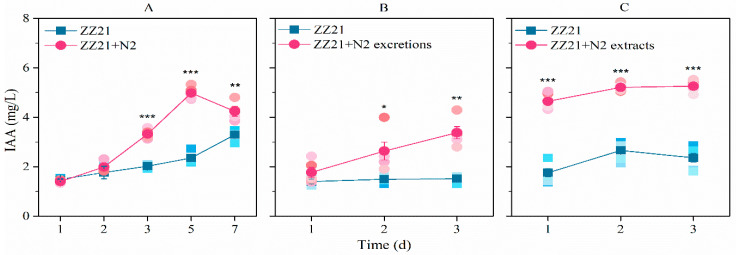
*C. elegans* N2 (**A**), their excretions (**B**) and their extracts (**C**) increase the ability of *A. pascens* ZZ21 to synthesize IAA. Asterisks indicate significant differences (* *p* < 0.05, ** *p* < 0.01, *** *p* < 0.001). Data are expressed as mean ± SE.

When nematodes were supplied to the medium, IAA production in *A. pascens* ZZ21 increased significantly, especially during the middle and late stages of cultivation (days 5 and 7) (Figure 3A), whereas the number of *C. elegans* increased to 232 worms/mL. Similarly to the effect of whole nematodes, excretions and extracts from *C. elegans* promoted IAA biosynthesis in *A. pascens* ZZ21 (Figure 3B,C). When excretions from *C. elegans* were added to the medium, the level of bacterial IAA production increased, with no significant effects on bacterial growth (Figure S1), suggesting that the increase in IAA content in the medium was not due to the presence of increased quantities of bacteria.

### 3.2. C. elegans Extracts Stimulate IAA Biosynthesis in A. pascens ZZ21 via the IPyA Pathway

**Figure 4:**
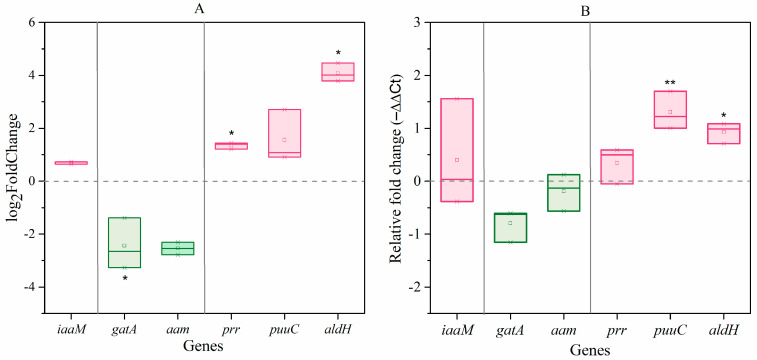
Effects of *C. elegans* extracts on the expression of genes involved in IAA biosynthesis in *A. pascens* ZZ21. (**A**) Results of transcriptome sequencing analysis (*p* < 0.05 and |log2(fold change)| > 1.5). (**B**) Results of qRT-PCR analysis. Asterisks indicate significant differences (* *p* < 0.05, ** *p* < 0.01).

Given the similar effects of *C. elegans* and their excretions or extracts on bacterial IAA biosynthesis, we used the extracts for further experiments. Transcriptome sequencing showed that the expression of 810 genes in *A. pascens* was significantly altered (*p* < 0.05 and |log2(fold change)| > 1.5) by treatment with *C. elegans* extracts; 252 genes were upregulated and 558 were downregulated. The expression of *prr* (ZZ21_GM003250) and *aldH* (ZZ21_GM001698), both encoding aldehyde dehydrogenase in the IPyA pathway, increased by 2.6-fold and 24.7-fold, respectively, in bacteria treated with *C. elegans* extracts compared to untreated control bacteria (Figure 4A). The expression of *gatA* (ZZ21_GM000216), which is predicted to encode an amidase in the IAM pathway, decreased by 75% in bacteria treated with *C. elegans* extracts compared to the control (Figure 4A). The results of qRT-PCR were similar to the results of transcriptome sequencing analysis (Figure 4B). These results demonstrate that *C. elegans* extracts have significant effects on the IPyA-IAA biosynthesis pathway. Finally, two genes (ZZ21_GM003237 and ZZ21_GM003887) encoding catalase, which functions in IAA degradation, were significantly downregulated in *A. pascens* ZZ21 treated with *C. elegans* extracts compared to the control, which may also result in increased bacterial IAA production.

Similar to the results of gene expression analysis, metabolomic analysis showed that the IPyA content in *A. pascens* ZZ21 treated with *C. elegans* extracts was ~1.7-fold greater than that in the control (Table 1). However, no significant changes were detected in the level of IAM, the major intermediate in the IAM pathway, in response to this treatment. These results further demonstrated that *C. elegans* extracts stimulate IAA biosynthesis in *A. pascens* ZZ21 via the IPyA pathway.

### 3.3. C. elegans Extracts Have Significant Effects on the Metabolism of A. pascens ZZ21

**Figure 5:**
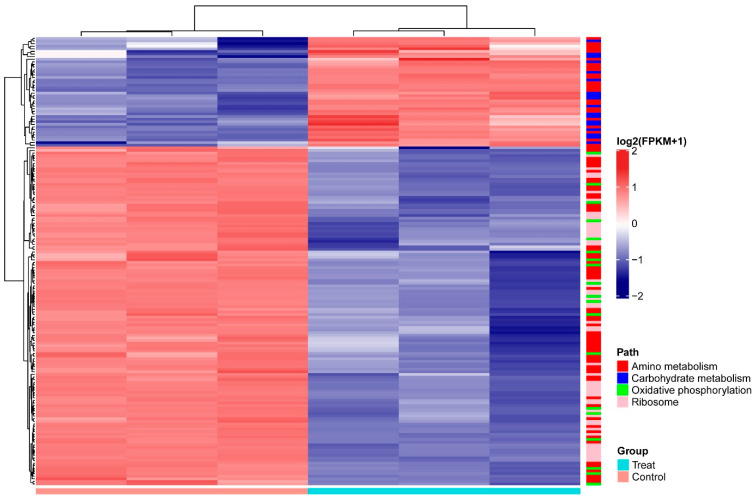
Heatmap of differentially expressed genes (*p* < 0.05 and |log2(fold change)| > 1.5) involved in amino metabolism, carbohydrate metabolism, oxidative phosphorylation and ribosomes of *A. pascens* ZZ21. Expression values (FPKM) were log_2_ transformed and normalized by Z-score. Genes that participate in different pathways are labeled with different colors to the right of the heatmap. *A. pascens* ZZ21 treated with *C. elegans* extracts was the experimental group (Treat), *A. pascens* ZZ21 without the treatment with *C. elegans* extracts was the control group (Control).

Among the genes differentially expressed in *A. pascens* ZZ21 in response to treatment with *C. elegans* extracts, we identified several genes related to metabolism in addition to those related to IAA biosynthesis. Based on KEGG analysis, 122 differentially expressed genes are involved in metabolism, including carbohydrate metabolism (18), energy metabolism (19) and amino acid metabolism (85). Furthermore, 50 genes involved in ribosome were downregulated in response to this treatment (Table 2 and Figure 5).

**Figure 6:**
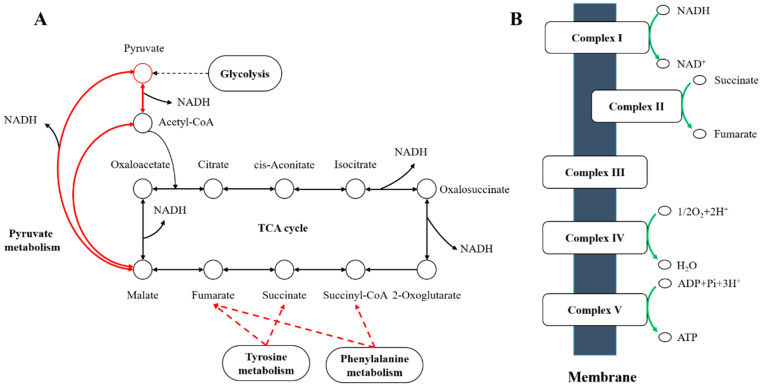
Sketch map of the effects of *C. elegans* extracts on the metabolism of *A. pascens* ZZ21. (**A**) Red arrows indicate metabolic processes promoted by the extract treatment, such as pyruvate metabolism, the citrate cycle (TCA cycle), and tyrosine and phenylalanine metabolism. (**B**) Green arrows indicate metabolic processes suppressed by the treatment, such as oxidative phosphorylation.

Among the differentially regulated carbohydrate metabolic pathways, genes encoding subunits of the pyruvate dehydrogenase complex, (ZZ21_GM003593, ZZ21_GM003355, ZZ21_GM003354, ZZ21_GM002702, ZZ21_GM001345, ZZ21_GM001346, ZZ21_GM002701, ZZ21_GM002703, ZZ21_GM001886 and ZZ21_GM001347), were significantly upregulated in the presence of *C. elegans* extracts. The gene (ZZ21_GM003611), encoding malate synthase, which catalyzes the synthesis of malate from acetyl-CoA, was also upregulated by this treatment, as was the gene encoding malate dehydrogenase (ZZ21_GM003610), which catalyzes the conversion between malate and pyruvate (Figure 6A). Although genes involved in pyruvate metabolism and the citrate cycle (TCA cycle) were upregulated in *A. pascens* ZZ21 in response to treatment with *C. elegans* extracts, genes involved in oxidative phosphorylation were downregulated by this treatment (Table 2 and Figure 6B). Genes involved in the electron transport chain were downregulated in the presence of *C. elegans* extracts, for example, genes encoding the subunits of electron transport chain complex I (ZZ21_GM001166), complex II (ZZ21_GM001142, ZZ21_GM001143 and ZZ21_GM001144), complex IV (ZZ21_GM002283, ZZ21_GM002284) and complex V (ZZ21_GM002743, ZZ21_GM002744, ZZ21_GM002745, ZZ21_GM002746, ZZ21_GM002747, ZZ21_GM002748, ZZ21_GM002749 and ZZ21_GM002750) (Figure 5).

Among genes associated with amino acid metabolism, most of those related to amino acid biosynthesis were downregulated in samples treated with *C. elegans* extracts (Figure 5). However, histidine, tyrosine and phenylalanine metabolism was promoted by the increased transcript abundance of genes related to these processes. Tyrosine and phenylalanine metabolism generate different metabolites that enter the TCA cycle (Figure 6). For example, the increased expression of genes (ZZ21_GM000266, ZZ21_GM002000, ZZ21_GM000957, ZZ21_GM002707, ZZ21_GM000265, ZZ21_GM003297 and ZZ21_GM000268), involved in tyrosine metabolism, promotes the biosynthesis of succinate and fumarate, which are intermediates in the TCA cycle (KEGG ID art00350, Figure 6A). Similarly, 14 genes involved in phenylalanine metabolism (ZZ21_GM003460, ZZ21_GM003461, ZZ21_GM003465, ZZ21_GM003472, ZZ21_GM003374, ZZ21_GM002000, ZZ21_GM003866, ZZ21_GM000957, ZZ21_GM003373, ZZ21_GM003372, ZZ21_GM003375, ZZ21_GM003297, ZZ21_GM003376 and ZZ21_GM002358) were strongly upregulated in the presence of *C. elegans* extracts, leading to increased levels of acetyl-CoA, fumarate and succinyl-CoA, which also enter into the TCA cycle (KEGG ID art00360, Figure 6A). Seven genes encoding proteins involved in histidine metabolism (KEGG ID art00340) were also significantly upregulated in response to *C. elegans* extracts, including genes encoding histidinol-phosphate aminotransferase (ZZ21_GM000957), histidine ammonia-lyase (ZZ21_GM000433, ZZ21_GM004017), urocanate hydratase (ZZ21_GM000878, ZZ21_GM000432), imidazolonepropionase (ZZ21_GM001297) and formiminoglutamase (ZZ21_GM000438). This results in the increased production of glutamate, which participates in alanine, aspartate and glutamate metabolism.

Metabolic analysis confirmed the effects of *C. elegans* extracts on *A. pascens* ZZ21 metabolism. Glucose-6-phosphate and pyruvic acid contents increased significantly in *A. pascens* ZZ21 cultured with *C. elegans* extracts vs. untreated controls (Table 1). These results are similar to the finding (from the transcriptome analysis) that genes involved in carbohydrate metabolism were upregulated in *A. pascens* ZZ21 in the presence of these extracts. Meanwhile, the levels of proline and a derivative of glycine (L-4-hydroxyphenylglycine) decreased in *A. pascens* ZZ21 in response to treatment with *C. elegans* extracts (Table 1), confirming that most genes involved in amino acid biosynthesis in *A. pascens* ZZ21 were suppressed by this treatment.

## 4. Discussion

### 4.1. C. elegans Metabolites Promote IAA Biosynthesis in A. pascens ZZ21

Understanding the extremely complex interactions between soil fauna and microorganisms is important in exploring how soil ecosystems function and maintain their stability [5,45,46]. In the current study, we demonstrated that the metabolites from bacterial-feeding nematodes accelerate IAA biosynthesis in IAA-producing bacteria without significantly altering the quantities of bacteria present (Figure 3 and Figure S1). This result is similar to the finding of Vandeputte et al. that treatment with leafy gall extracts significantly increased IAA production in *Rhodococcus fascians* strain D188-5 without meaningfully affecting the growth of the bacterium [47]. Our results indicate that in addition to feeding behavior, bacterial-feeding nematodes stimulate bacterial IAA biosynthesis by their non-feeding behavior (via secretory metabolites), providing new evidence to support the important roles of nematode metabolites in regulating the metabolism of their associated soil bacteria.

We used three different methods to identify the IAA production pathways in *A. pascens* ZZ21 that are affected by *C. elegans* extracts—qRT-PCR, transcriptomic analysis and metabolomic analysis. We observed that the expression of genes and the levels of characteristic intermediates involved in the IPyA-IAA biosynthesis pathway increased significantly in the presence of *C. elegans* extracts (Table 1 and Figure 4), indicating that the stimulatory effects of *C. elegans* extracts on IAA biosynthesis in *A. pascens* ZZ21 depend primarily on the IPyA pathway. Perhaps these extracts affected the expression of genes involved in the IPyA-IAA biosynthesis pathway because these genes are in general much more sensitive to the external environment than genes related to the IAM pathway. For example, *ipdC*, encoding indolepyruvate decarboxylase, a key enzyme in the IPyA pathway, is upregulated by osmotic and acid-induced stress [48,49]. Secondly, IAA and IAA-like compounds also induce the expression of *ipdC* [50,51], pointing to positive feedback regulation of IAA biosynthesis. Furthermore, IAA may induce a negative feedback in IAM-pathways. For example, the product IAA induces the expression of *ipdC* in a positive feedback manner in *Azospirillum brasilense* Sp245 [49]. On the other hand, both IAM and IAA inhibited the activity of tryptophan monooxygenase (TMO) enzyme, which catalyzes the conversion from Trp to IAM in *Pseudomonas savastanoi* [52]. In addition, *C. elegans* preys on *A. pascens* ZZ21; organic acids, amino acids and other ingredients (such as digestive enzymes) in *C. elegans* extracts may induce osmotic and predation stress in *A. pascens* ZZ21 [28,53]. Therefore, we speculate that the secretion of IAA may function as a predation defense strategy for *A. pascens* ZZ21, as IAA could help the bacterium better adapt to stress conditions (such as osmotic stress) by regulating the expression of genes related to survival, such as heat shock protein DnaK in *Escherichia coli* [54,55]. However, the mechanism by which metabolites from *C. elegans* increase the expression of genes related to IAA biosynthesis in *A. pascens* ZZ21 requires further study.

### 4.2. A. pascens ZZ21 Adjusts Its Metabolism to Defend Itself from Oxidative Stress Probably Induced by C. elegans

Although *A. pascens* ZZ21 perhaps resists predation pressure via IAA biosynthesis, the oxidative damage caused by predation and osmotic stress should not be underestimated [53,56]. Notably, auxin treatment and osmotic changes induce the formation of reactive oxygen species (ROS) in plants [57]. In this way, we speculate that the presence of *C. elegans* extracts may cause the production of ROS and oxidative damage. In spite of this, *A. pascens* ZZ21 regulated the expression of metabolism-related genes to help them deal with oxidative stress probably induced by *C. elegans* extracts. The increased expression of genes related to glycolysis, pyruvate metabolism, tyrosine and phenylalanine metabolism increased the production of reduced nicotinamide adenine dinucleotide (NADH). In addition, the overall downregulation of oxidative phosphorylation reduced the oxidation of NADH, thereby promoting its accumulation (Figure 6). In addition to its role in electron transfer via oxidative phosphorylation, NADH plays an important role in maintaining cellular redox homeostasis via antioxidant activity [58,59]. This finding suggests that *A. pascens* ZZ21 adjusts its metabolism to produce more NADH to decrease damage from oxidative stress induced by *C. elegans*.

On the other hand, we detected high concentrations of pyruvic acid and glucose-6-phosphate in the *A. pascens* ZZ21 metabolome in response to treatment with *C. elegans* extract (Table 1). Increased levels of these two compounds enhance TCA cycle flux by increasing the abundance of the substrates. This, in turn, increases the level of citric acid, which inhibits the activity of phosphofructokinase and further increases the level of glucose-6-phosphate. Glucose-6-phosphate enters into the pentose phosphate pathway, of which the major product is reduced nicotinamide adenine dinucleotide phosphate (NADPH) [60]. NADPH enhances the antioxidant capacity of the glutathione antioxidant system [61], which is also facilitated by histidine metabolism (Table 2). Furthermore, pyruvic acid inhibits hydrogen peroxide production directly via a non-enzymatic decarbonation reaction. These findings imply that *A. pascens* ZZ21 addresses oxidative damage by indirectly promoting NADPH biosynthesis, even though we did not detect any differentially expressed genes or metabolites directly related to the pentose phosphate pathway. Meanwhile, genes involved in protein and amino acid biosynthesis in *A. pascens* ZZ21 were downregulated by treatment with *C. elegans* extracts. This might explain why the growth of *A. pascens* ZZ21 was not altered during our experiment.

## 5. Conclusions

In conclusion, the results of this study indicate that *C. elegans* extracts promote IAA biosynthesis in *A. pascens* ZZ21 via the IPyA pathway, highlighting the important role of the excretions of bacterial-feeding nematodes in nematodes–bacteria interaction systems. *C. elegans* extracts also strongly affected the metabolism of *A. pascens* ZZ21. We suggest that *C. elegans* extracts caused stress to *A. pascens* ZZ21 and that IAA and its metabolic intermediates, such as pyruvic acid and NADH, play important roles in resisting the damage caused by this stress. Our findings indicate that secretory excretions of *C. elegans* played significant roles in mediating the interaction between nematodes and bacteria in the soil ecosystem, thus enriching our understanding of the interaction network of the soil ecosystem. Nevertheless, other mechanistic studies are needed to confirm our conclusion, such as the use of knock-out genes (or RNAi) of the IAA synthesis pathway in *A. pascens* to demonstrate the causal relationship of upstream metabolic genes/pathways and IAA production. In addition, further studies are needed to explore how the active ingredients in *C. elegans* extracts increase the expression of genes related to IPyA-IAA biosynthesis and how they influence the metabolism of *A. pascens* ZZ21.

## Figures and Tables

**Table 1:** Differential metabolites identified by metabolomic analysis ^a,b^.

	Metabolite	Log_2_(Fold Change)	*p* Value
**Upregulated metabolites**	Glycerol	2.68	0.02
	Pyrrole-2-carboxylic acid	2.59	0.03
	Trehalose-6-phosphate	1.63	0.00
	Vanillylmandelic acid	1.45	0.02
	Citramalic acid	1.29	0.00
	2-Amino-3-methyl-1-butanol	1.07	0.02
	Glucose-6-phosphate	0.93	0.01
	6-Phosphogluconic acid	0.89	0.03
	D-Altrose	0.82	0.00
	Lyxonic acid, 1,4-lactone	0.76	0.04
	Indole-3-pyruvic acid	0.74	0.01
	2,4-Diaminobutyric acid	0.61	0.01
	Pyruvic acid	0.60	0.00
**Downregulated metabolites**	Proline	−20.35	0.01
	Analyte	−3.82	0.02
	2-Amino-2-norbornanecarboxylic acid	−1.73	0.02
	Pipecolinic acid	−1.52	0.00
	D-Glyceric acid	−1.26	0.04
	l-4-Hydroxyphenylglycine	−1.19	0.01
	Galactinol	−0.96	0.00
	α-Ketoglutaric acid	−0.93	0.01
	Naringin	−0.86	0.00
	Allose	−0.83	0.02
	Quinolinic acid	−0.72	0.03
	Phytol	−0.63	0.05

^a^ Effects of *C. elegans* extracts on metabolites in the IAA biosynthesis pathway in *A. pascens* ZZ21. ^b^ *p* < 0.05, |log2(fold change)| > 0.6.

**Table 2:** Top enriched KEGG pathways of the differentially expressed genes.

	KEGG ID	KEGG Pathway	Number of Genes	*p* Value
**Upregulated genes**	art00360	Phenylalanine metabolism	14	2.62 × 10^−6^
	art00620	Pyruvate metabolism	17	0.002907
	art00020	Citrate cycle (TCA cycle)	12	0.004289
	art01120	Microbial metabolism in diverse environments	46	0.009110
	art00340	Histidine metabolism	7	0.014361
	art00350	Tyrosine metabolism	7	0.025357
**Downregulated genes**	art03010	Ribosome	50	1.68 × 10^−6^
	art01230	Biosynthesis of amino acids	61	0.002438
	art00190	Oxidative phosphorylation	19	0.004855
	art01210	2-Oxocarboxylic acid metabolism	15	0.030991
	art00290	Valine, leucine and isoleucine biosynthesis	10	0.039808

## Data Availability

Some or all data used during the study are available from the corresponding author by request.

## Supplementary Materials

The following are available online at https://www.mdpi.com/article/10.3390/microorganisms9050970/s1, Figure S1: Effects of *C. elegans* excretions on the growth of *A. pascens* ZZ21. Table S1: Primers used in this study.
