# Supplementary material for: Caenorhabditis elegans Extracts Stimulate IAA Biosynthesis in Arthrobacter pascens ZZ21 via the Indole-3-pyruvic Acid Pathway
Source: Microorganisms. 2021 Apr 30;9(5):970. doi: 10.3390/microorganisms9050970 (PMC8146544; doi:10.3390/microorganisms9050970)
Supplement: Supplementary file 1 [file microorganisms-09-00970-s001.zip › Table S1 Primers used in this study.pdf]

Table S1 Primers used in this study.

**Primer Name**	**Sequence 5′–3′ [a]**
iaaMF	CCTGGAAAGCCGCTGTGA
iaaMR	GCCGTAGAAGGTCTGCTCGTC
gatAF	CGAAACCACCATCCGCTACG
gatAR	TGGAACTGGCGAAGAAAGGC
aamF	GCAACATTGTCGGCTTCAGG
aamR	CGGACCGAAAGACCTGGG
prrF	ACTTCGGTCCGCTGAACAAC
prrR	CATCTCCACGGCTTCCTGTT
puuCF	GCCGCAGCAATCTCAAGC
puuCR	CCAGCAACGCAGCGAAGT
aldHF	GCAACACGGTGGTCTGGAAG
aldHR	GCCCAACAGGAACGGAACC
16sF	GGTTGCGATACTGTGAGGTG
