# Supplementary figures and images for: Caenorhabditis elegans Extracts Stimulate IAA Biosynthesis in Arthrobacter pascens ZZ21 via the Indole-3-pyruvic Acid Pathway
Source: Microorganisms. 2021 Apr 30;9(5):970. doi: 10.3390/microorganisms9050970 (PMC8146544; doi:10.3390/microorganisms9050970)

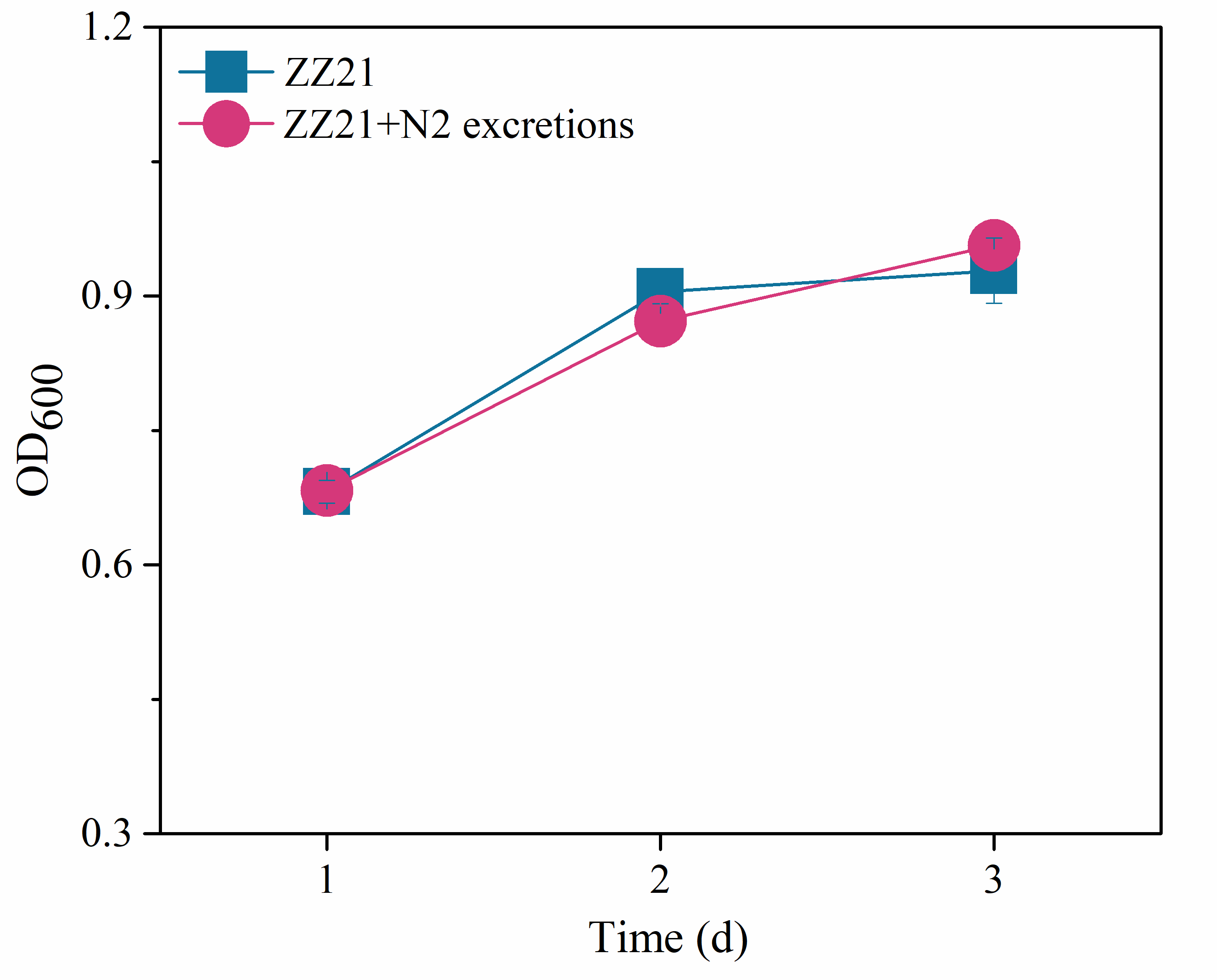

Supplement: Supplementary file 1 [file microorganisms-09-00970-s001.zip › Figure S1.tif]
